# Perivascular mast cells regulate vein graft neointimal formation and remodeling

**DOI:** 10.7717/peerj.1192

**Published:** 2015-08-18

**Authors:** Junxi Wu, Gianluca Grassia, Helen Cambrook, Armando Ialenti, Neil MacRitchie, Jaclyn Carberry, Roger M. Wadsworth, Catherine Lawrence, Simon Kennedy, Pasquale Maffia

**Affiliations:** 1 Strathclyde Institute of Pharmacy & Biomedical Sciences, University of Strathclyde, Glasgow, United Kingdom; 2 Centre for Immunobiology, Institute of Infection, Immunity and Inflammation, College of Medical, Veterinary and Life Sciences, University of Glasgow, United Kingdom; 3 Department of Pharmacy, University of Naples Federico II, Naples, Italy; 4 Institute of Cardiovascular and Medical Sciences, College of Medical, Veterinary and Life Sciences, University of Glasgow, United Kingdom

**Keywords:** Vein graft, Mast cells, Neointima

## Abstract

**Objective.** Emerging evidence suggests an important role for mast cells in vein graft failure. This study addressed the hypothesis that perivascular mast cells regulate *in situ* vascular inflammatory and proliferative responses and subsequent vein graft neointimal lesion formation, using an optimized local mast cell reconstitution method.

**Methods and Results.** Neointimal hyperplasia was induced by insertion of a vein graft into the right carotid artery in wild type and mast cell deficient Kit^W−sh/W−sh^ mice. In some experiments, mast cells were reconstituted systemically (tail vein injection of bone marrow-derived mast cells) or locally (directly into the right neck area) prior to vein grafting. Vein graft neointimal lesion formation was significantly (*P* < 0.05) reduced in Kit^W−sh/W−sh^ mice. Mast cell deficiency reduced the number of proliferating cells, and inhibited L-selectin, CCL2, M-CSF and MIP-3*α* expression in the vein grafts. Local but not systemic mast cell reconstitution restored a perivascular mast cell population that subsequently promoted neointimal formation in mast cell deficient mice.

**Conclusion.** Our data demonstrate that perivascular mast cells play a key role in promoting neointima formation by inducing local acute inflammatory and proliferative responses. These results suggest that *ex vivo* intraoperative targeting of mast cells may have therapeutic potential for the prevention of pathological vein graft remodeling.

## Introduction

Emerging evidence suggests that mast cells (MCs) play an active role in cardiovascular diseases (Kennedy et al., 2013; Kritikou et al., 2015) such as arteriosclerosis (Ihara et al., 1999; Kovanen, 2007; Sun et al., 2007a; Theoharides et al., 2001; Bot & Biessen, 2011; Bot, Shi & Kovanen, 2015; Wezel et al., 2015), aortic aneurysm (Sun et al., 2007b; Swedenborg, Mäyränpää & Kovanen, 2011; Zhang et al., 2012; Sillesen et al., 2015) and neointimal hyperplasia (Shiota et al., 1999; Fang et al., 2005; Jin et al., 2005; Jin et al., 2007). The presence of MCs in vein grafts has been known for decades; but only recently has the net contribution of these cells to vein graft disease been investigated. Earlier studies suggested an association between vein graft vasospasm and MC-derived histamine (Cross et al., 1988; Vural et al., 1999), while pharmacological inhibition of mast cell-derived chymase reduced neointimal hyperplasia in vein grafts (Jin et al., 2007). Recent evidence using mast cell-deficient Kit^W−sh/W−sh^ mice demonstrated a positive correlation between the presence of MCs and vein graft neointima thickening (De Vries et al., 2013). In that study, pharmacological inhibition of MCs also reduced vein graft hyperplasia with the complement factor C5a proposed to be an endogenous MC activator in vein graft disease (De Vries et al., 2013).

MCs can be reconstituted in c-Kit mutant mice by tail vein injection of wild type (WT) bone marrow-derived mast cells (BMMCs) (Grimbaldeston et al., 2005). In the present study, we demonstrate that correction of c-kit-mutation-induced MC deficiency in Kit^W−sh/W−sh^ mice by local but not systemic MC reconstitution restored neointimal formation to that seen in normal mice. Our results highlight the pivotal role played by perivascular MCs in promoting neointimal formation by inducing local early acute inflammatory and proliferative responses, and suggest vein graft *ex vivo* MC targeting as a potential therapeutic strategy to improve long-term vein graft patency.

## Materials and Methods

### Animals

Male wild type (WT) C57BL/6, either bred in-house (Biological Procedures Unit, University of Strathclyde) or purchased from Charles River (Margate, UK), and Kit^W−sh/W−sh^ mice (originally sourced from Jackson Laboratories, USA and subsequently bred in-house) were used in this study. All procedures on animals were performed in accordance with the United Kingdom Home Office Guide on the Use of Animals (Scientific Procedures) Act 1986 (Project Licence 60/4114). All mice were allowed free access to normal chow diet and water and maintained on a 12-hour light and dark cycle.

### Surgery

The mouse vein graft model used in this study was first established 17 years ago by Xu’s group (Zou et al., 1998) and well characterized for studying neointima hyperplasia over the past decade (Xu, 2004; Wu, Wadsworth & Kennedy, 2011). Briefly, mice were anesthetized with sodium pentobarbital (60 mg/kg, i.p.) with appropriate analgesic cover (buprenorphine; 0.05 mg/kg body weight, s.c.). Depth of anesthesia was assessed by loss of the pedal withdrawal reflex. The thoracic inferior vena cava was harvested from an isogenic donor mouse. The right common carotid artery of the recipient mouse was isolated and cut in the middle. A cuff made from a nylon tube was sleeved onto the distal arterial end. The artery was everted back over the cuff and ligated onto the cuff with 8/0 silk suture (Ethicon, Livingston, UK). The proximal arterial end was prepared in the same way. The vena cava segment was then sleeved onto the arterial ends in turn and fixed onto the cuff with 8/0 suture. At the study end point, mice were sacrificed by a rising concentration of CO_2_ unless otherwise specified and vein grafts harvested at 1–3, 7, 14 and 28 days after surgery.

### Bone marrow-derived mast cell (BMMC) reconstitution

Bone marrow cells were isolated from C57BL/6 mice and cultured in the presence of murine IL-3 and stem cell factor (PeproTech, New Jersey, USA) for 4 weeks as previously described (Sun et al., 2007a; Sun et al., 2007b). The phenotype of the BMMCs was confirmed by flow cytometry with anti-c-Kit and anti-FcεRI antibodies (eBioscience, Hatfield, UK). Systemic reconstitution of BMMCs is well established for restoration of a mast cell population in mast cell-deficient mice (Grimbaldeston et al., 2005). The recipient Kit^W−sh/W−sh^ mouse received 10 million wild type BMMCs via tail vein injection. Typically, BMMCs require around 9 weeks to migrate to peripheral tissues and re-establish a detectable MC population. However, in our study, although systemic reconstitution re-established mast cells in some organs, none were detected in perivascular locations. Consequently, we established a new method of local reconstitution by injecting BMMCs directly into the carotid artery region. Briefly, the recipient mouse was anesthetized and the common right carotid artery was carefully exposed. BMMCs were injected around the artery. The wound was closed with a 6/0 suture. Local reconstitution required only one million BMMCs to establish a perivascular mast cell population in a similar range to WT and this occurred within 4 weeks.

For all reconstitution experiments, interpositional vena cava to carotid artery vein grafting was performed as outlined above and grafts were removed 4 weeks after to measure neointima formation.

### Histology and immunostaining

Vein grafts were perfusion fixed and kept in 10% formalin overnight before embedding in paraffin. Serial sections (5 µm) were cut for each vein graft, and slides were evenly divided into 5 groups from the proximal to the distal end of each vein graft. For each staining experiment, a set of 5 slides (one slide from each group) from each vein graft was used. The mean value of the 5 serial slides was used to present the value of the whole vein graft. Haematoxylin and eosin (HE) staining was used for general morphology and planimetry studies (Image pro plus; Media Cybernetics, Marlow, UK).

Mast cells within grafts were identified and quantified by Toluidine Blue (Sigma-Aldrich, Dorset, UK) counterstained with fast red. MC identity was confirmed by Avidin Texas red conjugate staining (Invitrogen—Life Technologies, Paisley, UK). Avidin Texas red conjugate was added to de-waxed slides at a dilution of 1:250 in PBS and incubated overnight. Detection was by means of a fluorescence microscope (Biorad Radiance 2100 on a Nikon TE300) with 488 nm line of Argon ion laser excitation, emission 515 nm, and 543 nm line of Green He/Ne laser excitation, emission 590 nm.

For immunostaining, slides were de-waxed and pressure-cooked for antigen retrieval. As we previously demonstrated, cell proliferation peaks in the first week in mouse vein grafts (Wu, Wadsworth & Kennedy, 2011); therefore, proliferating cell nuclear antigen (PCNA) analysis was used to quantify neointimal proliferation of vein grafts 7 days after surgery (Grassia et al., 2009; Grassia et al., 2010) using a monoclonal mouse anti-PCNA antibody (1:200, PC10; Sigma-Aldrich, Dorset, UK) and biotin-SP-AffiniPure F(ab′)_2_ fragment goat anti-mouse IgG (H+L) secondary antibody (1:1000; Jackson ImmunoResearch Laboratories, West Grove, Pennsylvania, USA). Slides were treated with streptavidin–horseradish peroxidase (Dako, Ely, UK) and exposed to diaminobenzidine chromogen (Dako) with haematoxylin counterstain, as routinely performed in our lab (Grassia et al., 2009; Grassia et al., 2010). Nine sections from each vein graft were reviewed and scored under blind conditions. Data are presented as the percentage of cells positive for PCNA in the neointima 7 days after surgery.

### Assessment of pro-inflammatory markers in vein graft protein extract

The presence of pro-inflammatory markers in vein graft protein extracts at day 7 was assessed by using RayBio Mouse Atherosclerosis Antibody Array C (RayBiotech, Norcross, Georgia, USA) according to the manufacturer’s instructions. The array consisted of 22 different soluble signaling factor antibodies spotted in duplicate onto a PVDF membrane: fibroblast growth factor-basic (bFGF), CD40 (TNFRSF5), eotaxin-1 (CCL11), G-CSF, granulocyte-macrophage colony-stimulating factor (GM-CSF), IFN-*γ*, interleukin (IL)-1*α*, IL-1*β*, IL-2, IL-3, IL-4, IL-5, IL-6, IL-13, L-selectin (CD62L), monocyte chemoattractant protein-1 (MCP-1)(CCL2), macrophage colony-stimulating factor (M-CSF), macrophage inflammatory protein-3*α* (MIP-3*α*) (CCL20), P-selectin, RANTES (CCL5), tumor necrosis factor-*α* (TNF-*α*), and vascular endothelium growth factor-A (VEGF-A). Briefly, liquid nitrogen frozen vein grafts were crushed into powder and resuspended in 150 µL of high-salt extraction buffer (20 mM Hepes pH 7.9, 10 mM NaCl, 0.2 mM EDTA, 25% v/v glycerol, 0.5 mM phenylmethylsulfonyl fluoride, 1.5 µg/mL soybean trypsin inhibitor, 7 µg/mL pepstatin A, 5 µg/mL leupeptin, 0.1 mmol/L benzamidine, 0.5 mmol/L dithiothreitol) and incubated at 4 °C for 30 min with constant agitation. The samples were then centrifuged for 15 min at 6,000 g, and the supernatant aliquoted and stored at −80 °C. Protein concentration was determined using the Bio-Rad protein assay Kit. Total extracts were used to evaluate protein level by membrane-based antibody array. The membranes were blocked with 10% bovine serum albumin in PBS and subsequently incubated with lysates overnight at 4 °C. The membranes were washed with buffer supplied by the manufacturer, exposed to 500-fold diluted biotin-conjugated anti-cytokine antibodies at room temperature for 2 h, washed and incubated with a 1,000-fold diluted HRP-conjugated streptavidin for 1 h then immersed for 1 min in peroxidase substrate solution. The signal intensity of each spot was quantified by ImageJ (http://imagej.nih.gov/ij/). The signal intensity of the negative control was subtracted from each spot and the values were normalized to the positive controls. The resulted were expressed as Arbitrary Units.

### Serum cytokine detection assay

Concentration of bFGF, GM-CSF, IFN-*γ*, IL-1*α*, IL-1*β*, IL-2, IL-4, IL-5, IL-6, IL-10, IL-12, IL-13, IL-17, IFN *γ*-induced protein 10 (IP-10), KC (chemokine (C-X-C motif) ligand (CXCL) 1), MCP-1 (CCL2), monokine induced by IFN-*γ* (MIG), macrophage inflammatory protein-1*α* (MIP-1*α*), TNF-*α*, and VEGF were assessed in serum at day 7 using a 20-plex mouse cytokine assay (Macritchie et al., 2012; Sage et al., 2014) (Invitrogen—Life Technologies, Carlsbad, California, USA) and analyzed using a Bio-Rad Luminex 100 Plate Reader (Bio-Rad, Hemel Hempstead, United Kingdom).

### Characterization of leukocyte population by flow cytometry

Flow cytometry of vein graft tissue was performed as previously described with minor modifications (Macritchie et al., 2012; Hu et al., 2015). Briefly, 28 days after surgery WT and Kit^W−sh/W−sh^ mice were placed under terminal anesthesia and perfused with 2 mM EDTA (Sigma-Aldrich) in PBS via cardiac puncture to remove blood contamination from vascular tissue. Following removal of vein grafts, a single cell suspension was obtained by incubation of vein graft segments in an enzymatic suspension containing 450 U/ml collagenase type I, 125 U/ml collagenase type XI, 60 U/ml hyaluronidase, 60 U/ml DNase (all from Sigma-Aldrich) in PBS containing 20 mM Hepes at 37 °C for 1 h. Digested vein grafts were then mechanically disrupted through a 40 µM cell strainer to release a single cell suspension. Aliquots of cells were washed and resuspended in Fc block (2.4G2 hybridoma supernatant) for 25 min at 4 °C to block Fc receptors. Subsequently, cells were incubated with Abs (in PBS containing 2% FCS) for 30 min at 4 °C. Following washing, cells were analyzed on a MACSQuant Analyzer (Miltenyi Biotec, Bisley, UK) and data analysis performed using FlowJo (Tree Star Inc., Olten, Switzerland). Abs used were CD11b-PE-Cy7 (M1/70), CD11c-PE (N418) from eBioscience (Hatfield, UK), CD45-APC-Cy7 (30-F11), CD3-PerCP (145-2C11) from BD Biosciences (Oxford, UK), and B220-Brilliant Violet 421 (RA3-6B2) from Biolegend (Cambridge, UK). All the antibodies used for flow cytometry analysis of the mouse vein graft had been validated on cell suspensions from spleen/LNs untreated or treated with the enzyme digestion cocktail.

### Statistics

One-way ANOVA was performed for multi-group comparison, followed by Tukey’s test. Student’s *t*-test was applied for 2-group comparisons. In all cases, *P* < 0.05 was taken as statistical significance.

## Results

### Local rather than systemic MCs regulate vein graft neointimal hyperplasia

**Figure 1:**
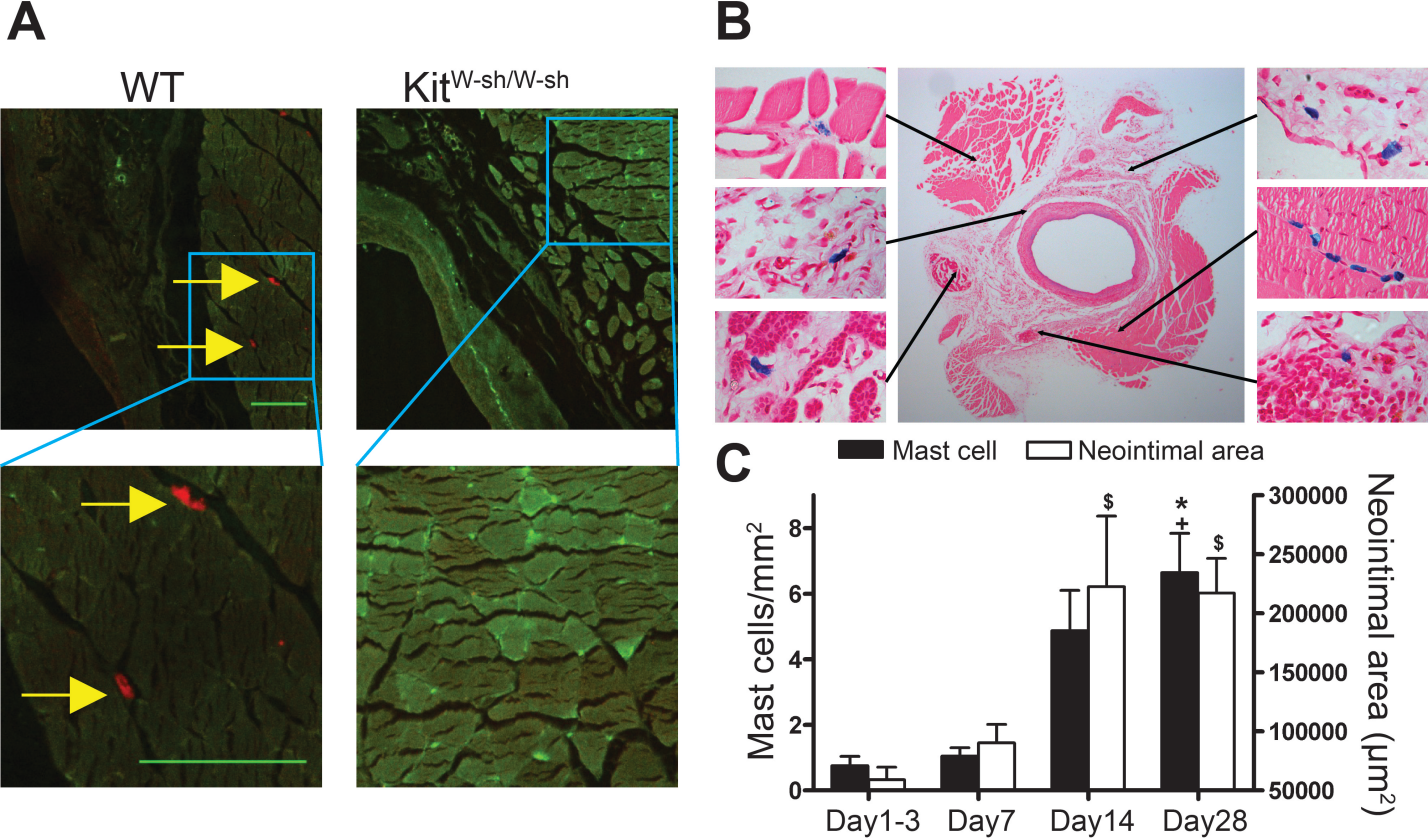
Perivascular accumulation of MCs in vein graft correlates with neointima formation. MC anatomical distribution in vein grafts was assessed using (A) Texas Red-Avidin staining (arrows indicate mast cells; Bar = 100 µm) or (B) Toluidine Blue staining, showing perivascular MC accumulation in WT vein grafts 28 days following surgery. (C) MC number increased in vein grafts and correlated with neointima formation up to 4 weeks (*n* = 4–9). *Y* axis on the left represents mast cell density, ^∗^*P* < 0.05 vs. day 1–3; +*P* < 0.05 vs. day 7. *Y* axis on the right represents neointima area: $*P* < 0.05 vs. day 1–3. Error bars show the standard error of the mean. Data are analyzed by one-way ANOVA with Tukey’s post hoc test.

MCs were present in the adventitia of vein grafts from control WT mice (Figs. 1A and 1B). In Kit^W−sh/W−sh^ mice, no MCs were found in either healthy arteries (not shown) or vein grafts (Fig. 1A). Interestingly, in WT mice perivascular MC count was low 1–3 days following surgery and gradually increased up to 4 weeks, in parallel with neointimal thickening (Fig. 1C).

**Figure 2:**
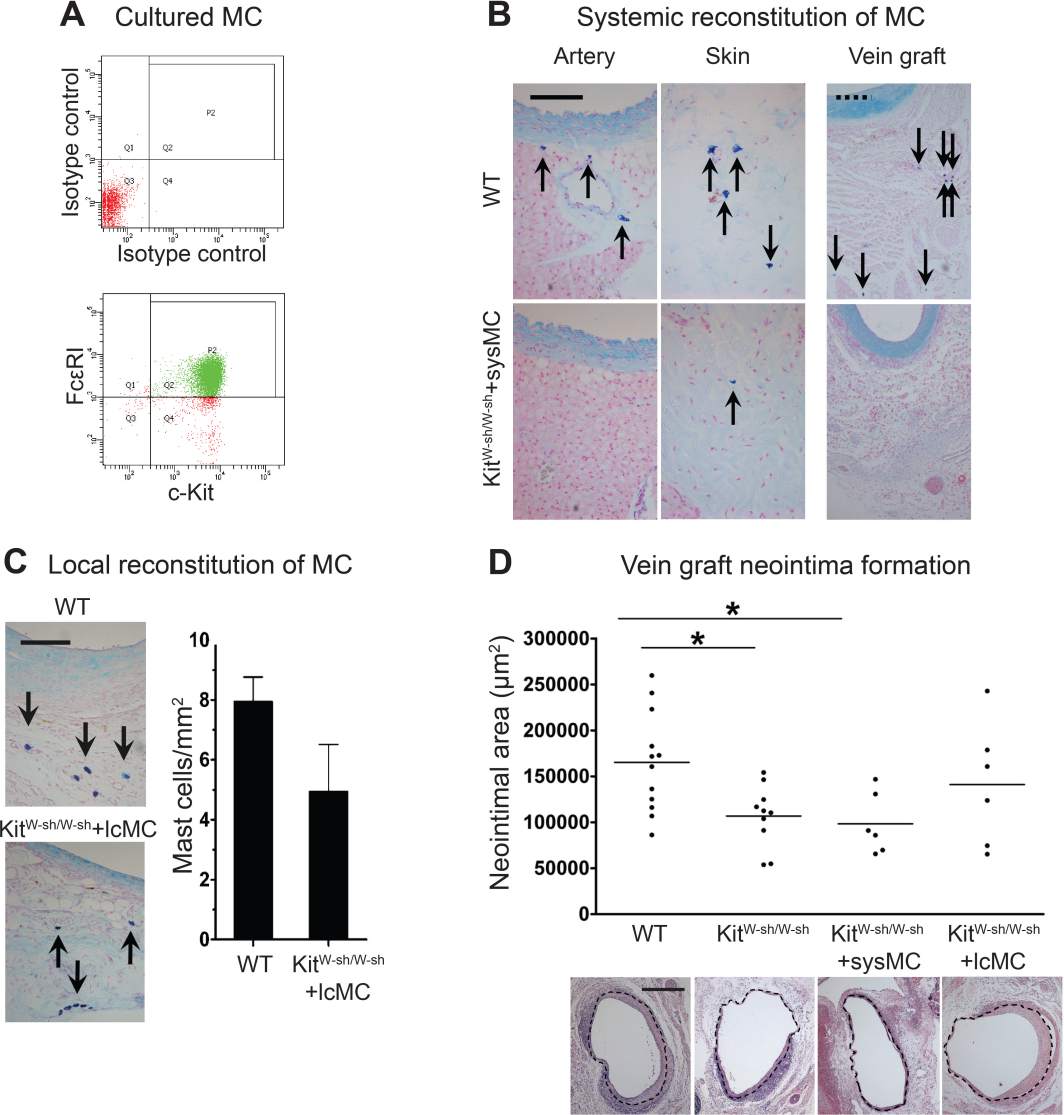
Reconstitution of WT mast cells to Kit^W−sh/W−sh^ mice. (A) WT BMMCs were cultured and characterized by flow cytometry with anti-FcεRI and anti-c-Kit antibodies. 90% of the living cells were positive for both FcεRI and c-Kit. (B) Systemic administration of BMMCs via tail vein achieved multiple organ reconstitution (skin shown in figure), however, no MCs were detected in the vascular wall of the aorta or in the vein graft 28 days after surgery (Solid bar in left panels = 100 µm; dashed bar in right panels = 200 µm). (C) Local reconstitution restored mast cells in Kit^W−sh/W−sh^ vein grafts (Bar = 100 µm). No mast cells were found in any other organ or tissue. Data corresponds to twelve mice per group (WT) and six mice per group (Kit^W−sh/W−sh^+lcMC). Error bars show the standard error of the mean. No statistical difference was detected between groups by Student’s *t*-test. (D) MCs increase neointimal formation in vein grafts. Upper panel: the neointimal area of 28-day-old vein grafts from wild type (WT) mice, Kit^W−sh/W−sh^ mice with or without systemic (Kit^W−sh/W−sh^ +sysMC) or local (Kit^W−sh/W−sh^ +lcMC) MC reconstitution. Individual data points represent average value per mouse; horizontal bars denote mean. ^∗^*P* < 0.05 by one-way ANOVA with Tukey’s post hoc test. Lower panel: representative pictures (H&E) of cross sections of vein graft middle portion from respective groups. The dashed circle indicates the border of neointima. (Bar = 500 µm).

At 28 days following surgery, MC deficient Kit^W−sh/W−sh^ mouse vein grafts had developed significantly less neointima when compared to control WT mice (1.07 × 10^5^ ± 0.11 × 10^5^ vs. 1.65 × 10^5^ ± 0.16 × 10^5^ µm^2^, respectively, *P* < 0.05; Fig. 2D); confirming previous findings by De Vries et al. (2013). Importantly, Kit^W−sh/W−sh^ mice carrying an inversion mutation in the c-Kit promoter region (Grimbaldeston et al., 2005) present other Kit-related abnormalities (Kennedy et al., 2013); therefore, reconstitution of these mutant mice with bone marrow-derived mast cells is required to selectively restore MC populations, and to confirm the MC-specificity of the observed neointimal reduction (Sun et al., 2007a; Sun et al., 2007b; Kennedy et al., 2013). Thus, prior to vein graft implantation, some Kit^W−sh/W−sh^ mice received MC reconstitution via systemic or local injection. The phenotype of the BMMCs was confirmed by flow cytometry with anti-c-Kit and anti-Fcε RI antibodies. Over 90% of the cells were double positive for both markers by 4 weeks (Fig. 2A). Consistent with previous reports (Grimbaldeston et al., 2005), systemic injection achieved multi-organ MC reconstitution (skin shown in Fig. 2B). However, no MCs were detected in vein grafts or in the aorta 28 days after surgery (Fig. 2B). Conversely, local injection successfully introduced a MC population around the vein graft at 28 days (Fig. 2C), but not elsewhere (not shown). Intriguingly, local MC reconstitution, but not systemic reconstitution, restored the amount of neointima formation to that observed in WT mice grafts (1.41 × 10^5^ ± 0.27 × 10^5^ vs. 1.65 × 10^5^ ± 0.16 × 10^5^ µm^2^, respectively; Fig. 2D). These data suggest a critical role for the perivascular MCs in neointimal formation and vein graft disease.

### Perivascular MCs up-regulated acute inflammation and promoted cell proliferation

**Figure 3:**
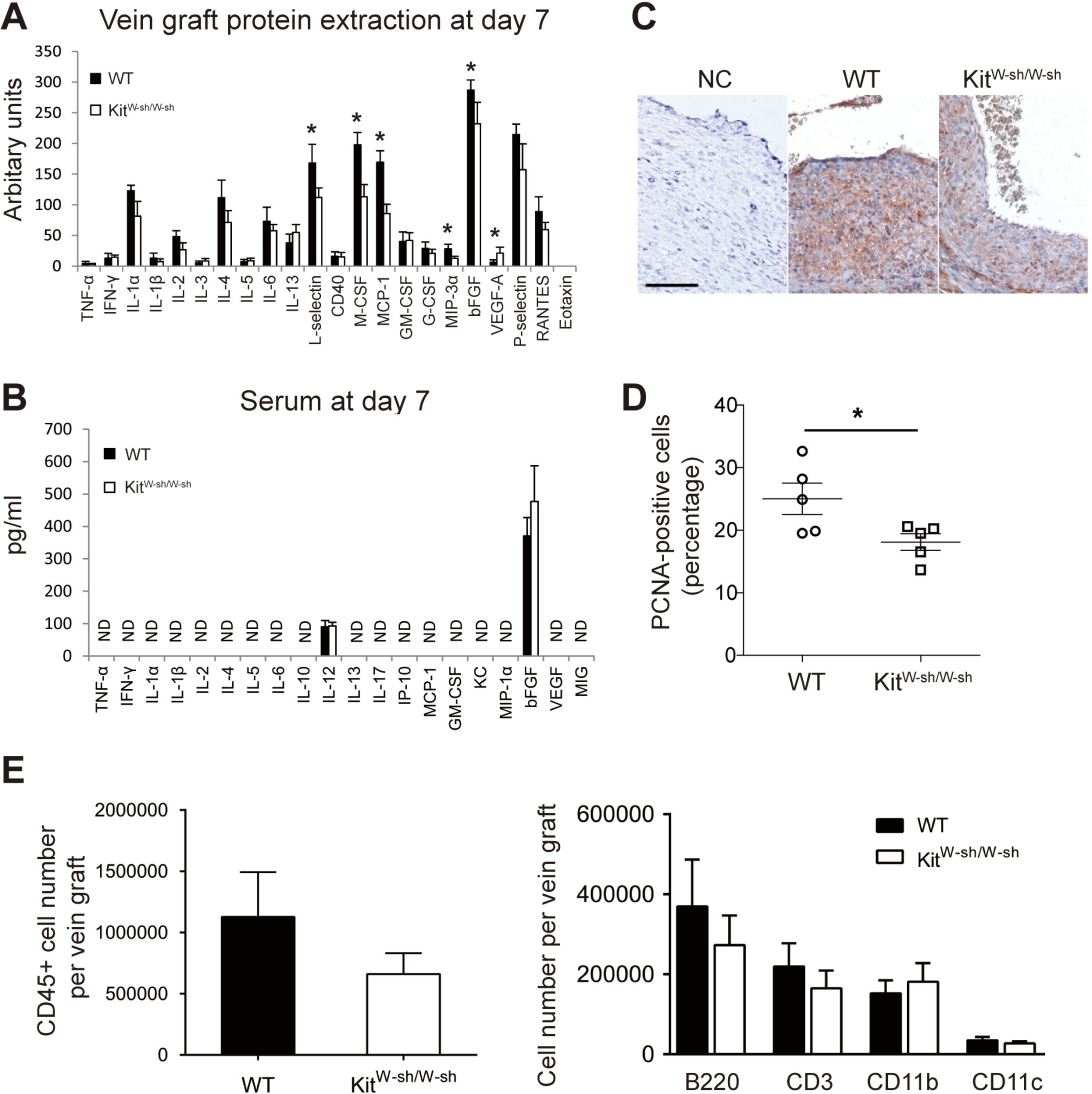
MCs increase vascular inflammatory and proliferative response. Cytokine expression profile in vein graft protein extracts (A) and serum (B) at day 7. ND, Not detectable. Error bars show the standard error of the mean. ^∗^*P* < 0.05 by unpaired Student’s *t*-test (*n* = 5 for vein graft samples and *n* = 9–10 for serum samples). (C) Proliferating cells in vein grafts at day 7 were identified by anti-PCNA staining. Sections incubated with the secondary antibody alone were used as negative control (NC). (Bar = 100 µm). (D) The percentage of proliferating cells was quantified and data presented as mean ± SEM. ^∗^*P* < 0.05 by unpaired Student’s *t*-test. (E) The total leukocytes (CD45) as well as the subsets of B cells (B220), T cells (CD3), CD11b+ve, and CD11c+ve cells in the WT and Kit^W−sh/W−sh^ vein grafts were quantified by flow cytometry at 28 days. Data correspond to five mice per group. Error bars show the standard error of the mean. No statistical difference was detected between groups by Student’s *t*-test.

At 7 days, an elevated expression of pro-inflammatory markers was detectable in WT mouse vein grafts compared to MC deficient mice grafts (Fig. 3A). In particular, the significantly higher levels of L-selectin (*P* < 0.05), MCP-1/CCL-2 (*P* < 0.05), M-CSF (*P* < 0.05) and MIP-3*α* (*P* < 0.05) observed in WT grafts may increase adhesion and recruitment of circulating leukocytes and promote cell proliferation. Importantly, this pattern was not replicated in serum samples (Fig. 3B), once again supporting the hypothesis that MCs control the local vascular pro-inflammatory response, but exert no systemic effect.

There is strong evidence for an important role for CCL-2 and M-CSF in vascular smooth muscle cell proliferation, neointimal formation and vein graft thickening (Selzman et al., 2002; Schepers et al., 2006; Shiba et al., 2007; Grassia et al., 2009; Fu et al., 2012). We therefore assessed cell proliferation in mouse vein grafts at day 7, the peak of cell proliferation in this model as previously demonstrated (Wu, Wadsworth & Kennedy, 2011), by anti-PCNA staining. WT vein grafts were found to have a higher percentage of proliferating cells compared to MC deficient mice (*P* < 0.05; Figs. 3C–3D). These data demonstrate the key role played by perivascular MCs in orchestrating the inflammatory-proliferative response leading to accelerated neointima formation in vein graft disease. Interestingly, no significant difference was observed in vein graft leukocyte composition between MC deficient and WT mice at 28 days (Fig. 3E). Data supporting a key role played by the acute vascular inflammatory/proliferative response only during the first 14 days following surgery.

## Discussion

Our data demonstrate a detrimental role played by local perivascular MCs in vein graft neointimal formation. MC perivascular number correlated with increased neointimal formation following vein graft surgery. We confirmed that MC deficiency reduced vein graft neointimal formation, as recently demonstrated by De Vries et al. (2013). Additionally, we demonstrated that correction of MC deficiency in Kit^W−sh/W−sh^ mice by local but not systemic MC reconstitution restored the reduced neointimal formation. Our results highlight the importance of the local effects of perivascular MCs in the development of vein graft disease and form the basis for further investigation on vein graft MC *ex vivo* manipulation as a potential therapeutic strategy to improve long-term vein graft patency.

It was not surprising that tail vein injection failed to reconstitute perivascular MCs in healthy Kit^W−sh/W−sh^ mice. Sun et al. (2007a) and Sun et al. (2007b) found that systemic reconstitution of perivascular MCs could be achieved only in pro-atherosclerotic Kit^W−sh/W−sh^/low density lipoprotein receptor deficient (Ldlr^−/−^) mice fed high fat diet, but not in the same mouse strain fed normal chow diet. Later studies showed that perivascular MC recruitment was a CCR2-dependent inflammatory process (Zhang et al., 2012). In contrast, local MC reconstitution was successful in eliminating the difference in neointimal area between WT and Kit^W−sh/W−sh^ mice.

We have previously demonstrated that neointimal formation in the mouse WT vein graft model is preceded by acute vascular inflammation and cell infiltration and proliferation during the first 14 days following surgery (Wu, Wadsworth & Kennedy, 2011). Intriguingly, our current mechanistic analysis demonstrates that adventitial/perivascular MCs play a pivotal role in orchestrating this early local acute inflammatory and proliferative response. In particular, MCs promoted vascular inflammation by increasing the expression of chemotactic factors (CCL-2, MIP-3*α*), cytokines (M-CSF) and cell adhesion molecules (L-selectin) in the vein graft, all of which, in turn, promote neointimal formation. Intriguingly, MC deficiency also reduced the levels of bFGF in the vein graft. Controlled release of bFGF has been reported to improve vein graft long-term patency (Haraguchi et al., 2007); however, the high circulating levels of bFGF observed in both MC WT and deficient mice could contribute to the neutralization of any potential detrimental effect due to the bFGF reduction in MC deficient grafts.

In order to further define the role that mast cells are playing additional experiments would be required to assess if the correction of MC deficiency in Kit^W−sh/W−sh^ mice by local MC reconstitution would be able to restore a proliferative/inflammatory response in the vein graft to the same level observed in WT mice. In addition, it would be important to assess which proliferating cells are affected by the MC deficiency.

## Conclusions

In conclusion, our data provide new insight into the role of perivascular MCs in the regulation of vein graft neointima formation. Coronary artery bypass grafting (CABG) remains the treatment of choice for high-risk coronary heart disease patients (Serruys et al., 2009); however, CABG patency is an issue in the long-term (Goldman et al., 2004) and there is a clear need for novel therapeutic strategies to address CABG graft failure (Bradshaw & Baker, 2013). We acknowledge that our animal studies require judicious interpretation when extrapolating to human disease, but our results may form the basis for testing new specific intra-operative graft *ex vivo* treatments based on MC targeting in the prevention of vein graft failure.

## Supplemental Information

10.7717/peerj.1192/supp-1 Supplemental Information 1 Supplemental raw data
