# Supplementary material for: Perivascular mast cells regulate vein graft neointimal formation and remodeling
Source: PeerJ. 2015 Aug 18;3:e1192. doi: 10.7717/peerj.1192 (PMC4548472; doi:10.7717/peerj.1192)
Supplement: Supplemental Information 1 [file peerj-03-1192-s001.docx]

**Supplemental Raw data**

**Perivascular mast cells regulate vein graft neointimal formation and remodeling**

Junxi Wu, Gianluca Grassia, Helen Cambrook, Armando Ialenti, Neil MacRitchie, Jaclyn Carberry, Roger M Wadsworth, Catherine Lawrence, Simon Kennedy and Pasquale Maffia


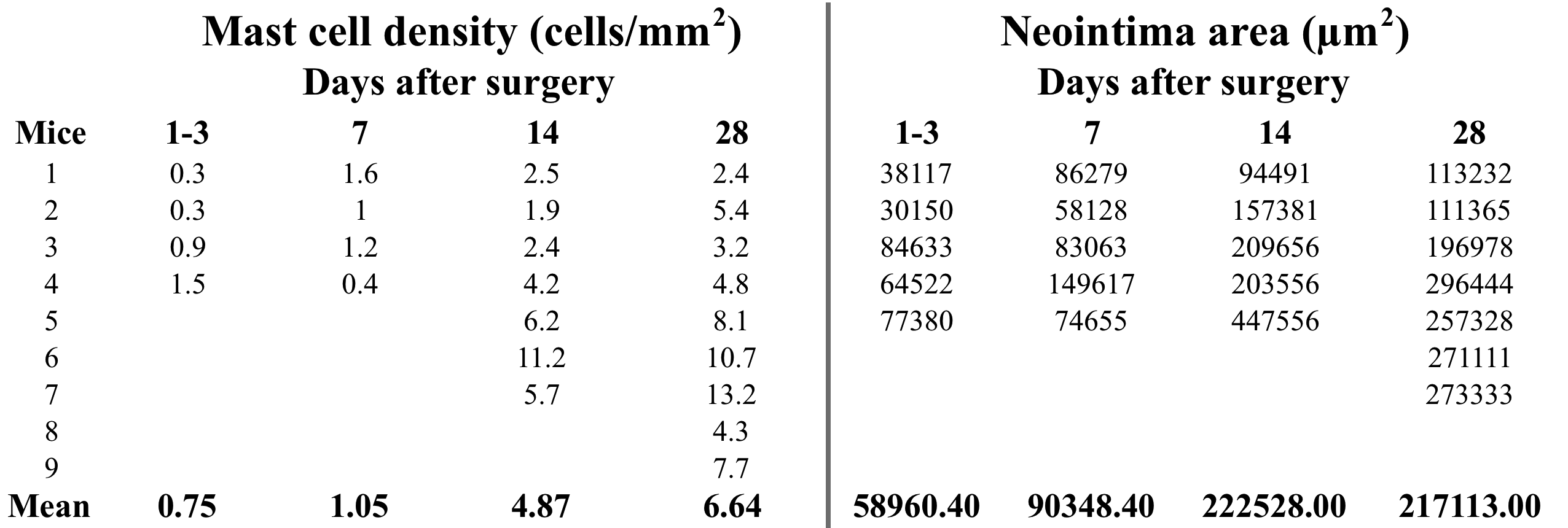


**Supplemental raw data Figure 1C**

Male wild type (WT) C57BL/6 mice were sacrificed at 1-3, 7, 14, and 28 days after vein graft surgery. The vein grafts were harvested, perfused, fixed and embedded in paraffin. Serial sections (5 μm) were cut for each vein graft, and slides were divided into 5 groups from the proximal to the distal end of each vein graft. For each staining experiment, a set of 5 slides (one slide from each group) from each vein graft was used. Haematoxylin and eosin (HE) staining was used for the neointima area quantification. Mast cells within grafts were identified by Toluidine Blue and expressed as number of mast cells per area. The mean values of the 5 serial slides are reported in the tab.


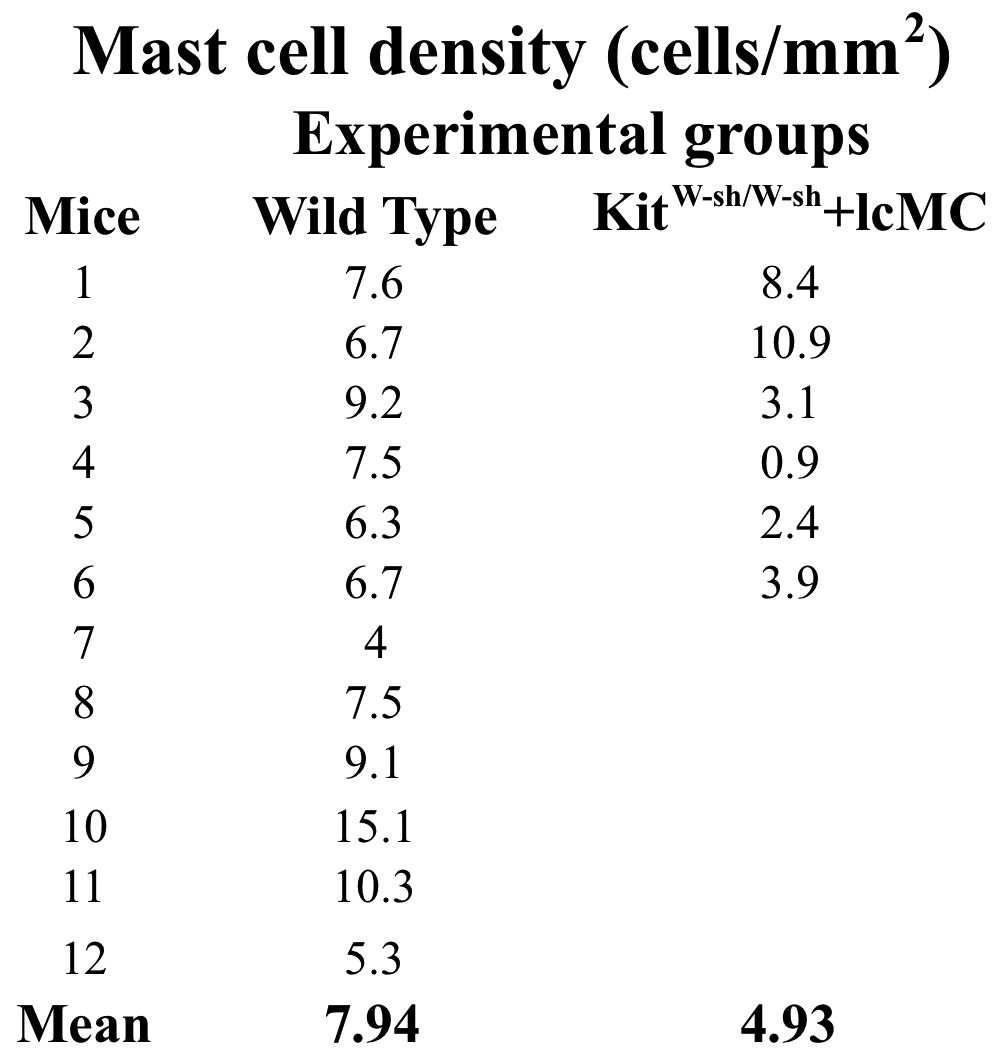


**Supplemental raw data Figure 2C**

Twenty-eight days after surgery, mast cells within grafts were identified by Toluidine blue counterstained with fast red, counted in the total section and expressed as number of mast cells per area. The data reported in the table are the mean values of 5 serial sections per animal. Kit^W-sh/W-sh^+lcMC = local reconstitution.


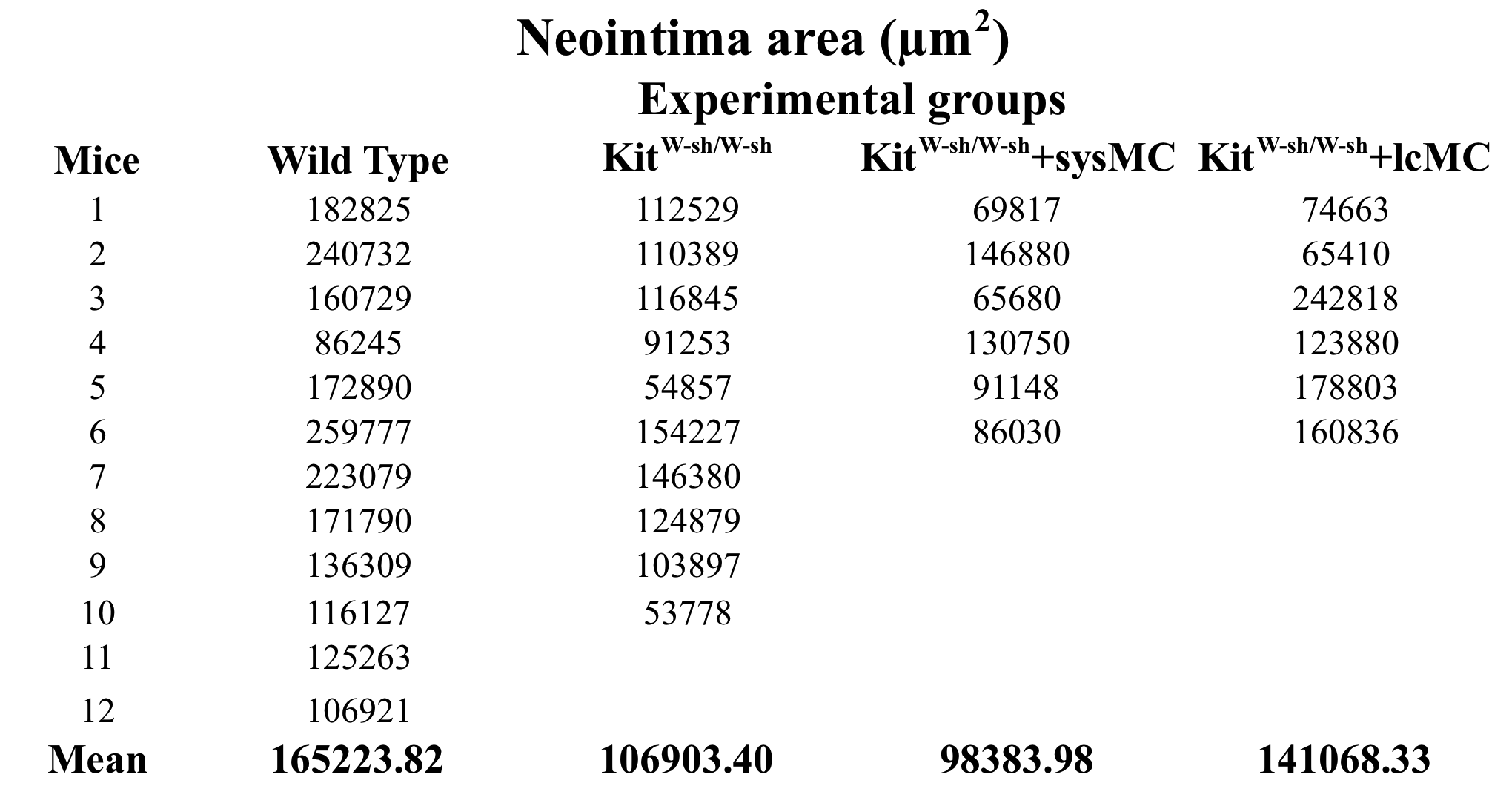


**Supplemental raw data Figure 2D**

Twenty-eight days after surgery the vein grafts were harvested. Five serial sections per mice were stained with HE and used for the neointima area quantification. The data reported in the table are the mean values of 5 serial sections per animal. Kit^W-sh/W-sh^+lcMC = local reconstitution; Kit^W-sh/W-sh^+sysMC = systemic reconstitution.


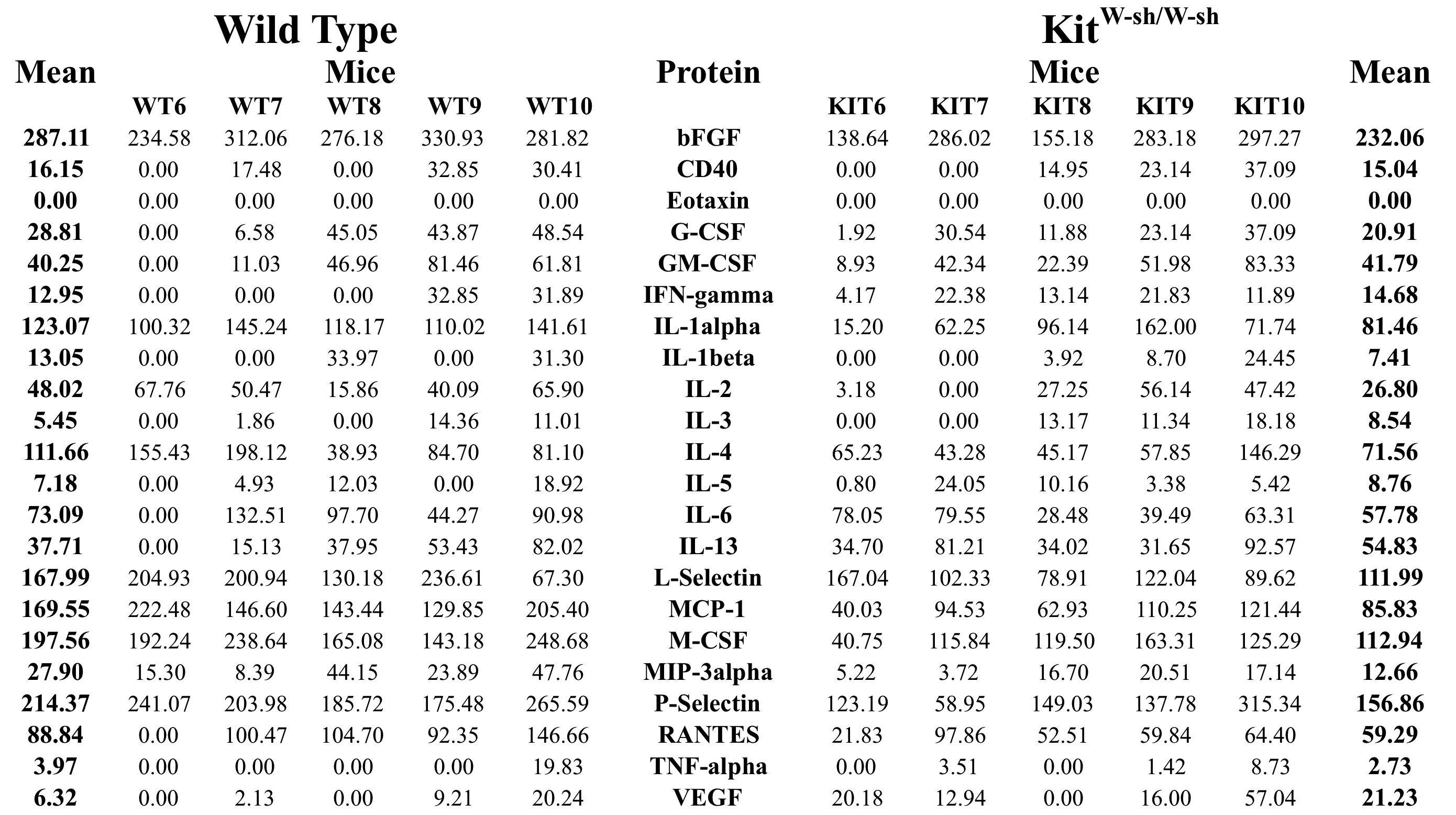


**Supplemental raw data Figure 3A**

For the assessment of pro-inflammatory markers in vein graft protein extracts, the vein grafts were harvested 7 days after surgery. Ten vein grafts (n=5 for wild type and Kit^W-sh/W-sh^) were crushed into powder using liquid nitrogen and treated according to the kit instructions provided (Mouse Atherosclerosis Array, Raybiotech). The values (arbitrary units) reported in the table are the means of two measurements normalized to the highest positive control value present on the membranes.


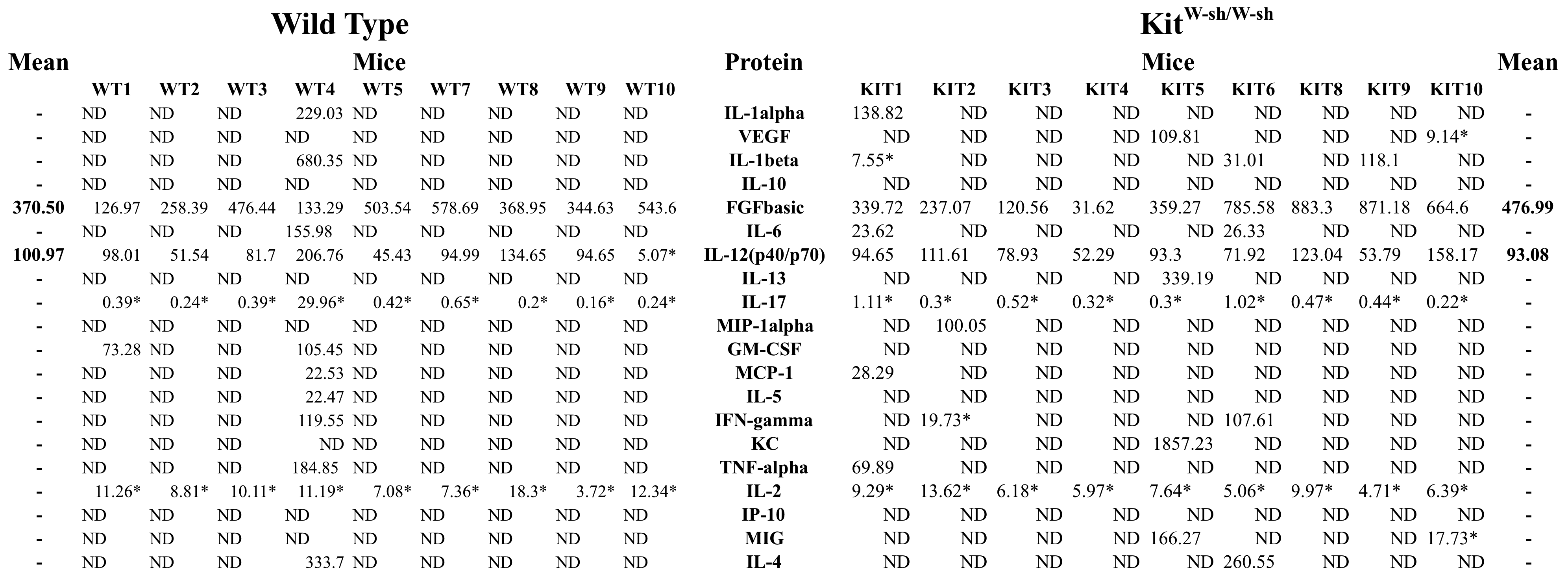


**Supplemental raw data Figure 3B**

Cytokine expression profile was assessed in serum at day 7 using a 20-plex mouse cytokine assay (Invitrogen - Life Technologies) and analyzed using a Bio-Rad Luminex. In table are reported the mean values of three replicates for each animal. ND=Not detectable. *Value = Value extrapolated beyond standard range.


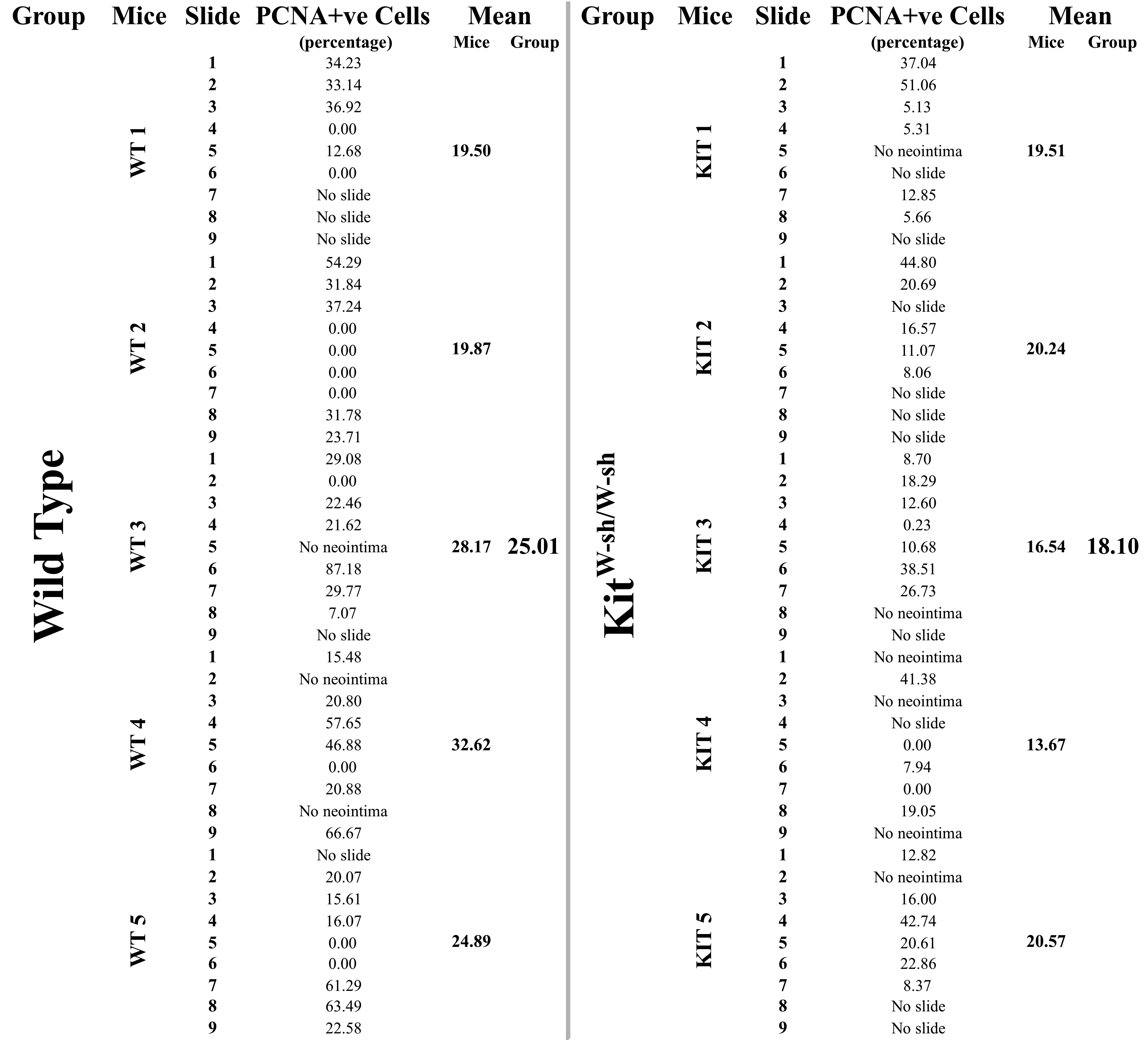


**Supplemental raw data Figure 3D**

For the PCNA immunostaining, the vein grafts were harvested 7 days after surgery. Nine serial sections per sample were analyzed. The number of PCNA positive cells (PCNA+ve) and the number of total cells were counted per each section, and the results expressed as percentage of cells positive for PCNA in the neointima 7 days after surgery. In the table the percentage values per each single section, mouse and group are reported.

**Supplemental raw data Figure 3E**

The total leukocytes (CD45) as well as the subsets of B cells (B220), T cells (CD3), CD11b+ve, and CD11c+ve cells in the WT and Kit^W-sh/W-sh^ vein grafts were quantified by flow cytometry at 28 days. In the table the numbers of positive cells for each marker, mouse and group are reported.
